# Impact of Left Atrial Bipolar Electrogram Voltage on First Pass Pulmonary Vein Isolation During Radiofrequency Catheter Ablation

**DOI:** 10.3389/fphys.2020.594654

**Published:** 2020-12-15

**Authors:** Lohit Garg, Naga Venkata K. Pothineni, J. Michael Daw, Matthew C. Hyman, Jeffrey Arkles, Cory M. Tschabrunn, Pasquale Santangeli, Francis E. Marchlinski

**Affiliations:** Electrophysiology Section, Cardiovascular Division, Hospital of the University of Pennsylvania, Philadelphia, PA, United States

**Keywords:** first pass isolation, voltage mapping, atrial fibrillation, pulmonary vein isolation, atriopathy

## Abstract

**Background:**

First pass pulmonary vein isolation (PVI) is associated with durable isolation and reduced recurrence of atrial fibrillation (AF).

**Objective:**

We sought to investigate the relationship between left atrial electrogram voltage using multielectrode fast automated mapping (ME-FAM) and first pass isolation with radiofrequency ablation.

**Methods:**

We included consecutive patients (pts) undergoing first time ablation for paroxysmal AF (pAF), and compared the voltage characteristics between patients with and without first pass isolation. Left atrium (LA) adjacent to PVs was divided into 6 regions, and mean voltages obtained with ME-FAM (Pentaray, Biosense Webster) in each region and compared. LA electrograms with marked low voltage (<0.5 mV) were identified and the voltage characteristics at the site of difficult isolation was compared to the voltage in adjacent region.

**Results:**

Twenty consecutive patients (10 with first pass and 10 without) with a mean age of 63.3 ± 6.2 years, 65% males, were studied. Difficult isolation occurred on the right PVs in eight pts and left PVs in three pts. The mean voltage in pts without first pass isolation was lower in all 6 regions; posterior wall (1.93 ± 1.46 versus 2.99 ± 2.19; *p* < 0.001), roof (1.83 ± 2.29 versus 2.47 ± 1.99; *p* < 0.001), LA-LPV posterior (1.85 ± 3.09 versus 2.99 ± 2.19, *p* < 0.001), LA-LPV ridge (1.42 ± 1.04 versus 1.91 ± 1.61; *p* < 0.001), LA-RPV posterior (1.51 ± 1.11 versus 2.30 ± 1.77, *p* < 0.001) and LA-RPV septum (1.55 ± 1.23 versus 2.31 ± 1.40, *p* < 0.001). Patients without first pass isolation also had a larger percentage of signal with an amplitude of <0.5 mV for each of the six regions (12.8% versus 7.5%). In addition, the mean voltage at the site of difficult isolation was lower at 8 out of 11 sites compared to mean voltage for remaining electrograms in that region.

**Conclusion:**

In patients undergoing PVI for paroxysmal AF, failure in first pass isolation was associated with lower global LA voltage, more marked low amplitude signal (<0.5 mV) and lower local signal voltage at the site with difficult isolation. The results suggest that a greater degree of global and segmental fibrosis may play a role in ease of PV isolation with radiofrequency energy.

## Introduction

Pulmonary vein isolation (PVI) with catheter ablation is an effective therapy for paroxysmal atrial fibrillation (pAF) and is recommended for drug refractory symptomatic pAF (January et al., 2014). Durability of PVI is important and reconnection of previously isolated pulmonary veins (PVs) is associated with higher risk of recurrent AF (Callans et al., 2004; Lemola et al., 2004; Nilsson et al., 2006; Natale et al., 2010; January et al., 2014). There are several factors responsible for reconnection of pulmonary veins, including non-contiguous lesions, transmurality of lesion, and presence of scar based on electroanatomic mapping. In patients undergoing ablation for pAF, a left atrial scar can be reproducibly identified in the sinus rhythm using a voltage range of 0.2–0.45 mV, correlating with delayed enhancement abnormalities identified on cardiac magnetic resonance imaging (Sanders et al., 2003; Verma et al., 2005; Kapa et al., 2014; Squara et al., 2014). Previous studies have reported that in patients with pAF, there is wide range (2–15%) of low voltage (<0.5 mV) areas (Anter et al., 2015; Liang et al., 2017). Some reports suggest that the left atrium with a bipolar voltage between 0.5 and 1.3 mV can also be considered as a transitional zone with moderate atrial fibrosis (Lin et al., 2014; Saini et al., 2017). First pass isolation during initial catheter ablation has been shown to be associated with durable PVI and a lower risk of recurrence (Sandorfi et al., 2018; Yamaguchi et al., 2020). The impact of presence of low electrogram voltage on first pass isolation has not been investigated. In this study, we aimed to determine the relationship between the left atrial bipolar electrogram voltage obtained with multielectrode fast automated mapping (ME-FAM) in sinus rhythm and first pass isolation with radiofrequency catheter ablation.

## Materials and Methods

### Study Population

We identified consecutive patients who underwent first time ablation with radiofrequency for symptomatic pAF with a standard ablation technique. Patients with complete ME-FAM performed in sinus rhythm were included. Patient demographics, clinical and procedural characteristics were assessed. All patients provided written informed consent for both the ablation procedure we well as inclusion of demographic information and procedural data in a medical research registry that was approved by the University of Pennsylvania Health System’s Institutional Review Board.

### EAM Technique

**FIGURE 1:**
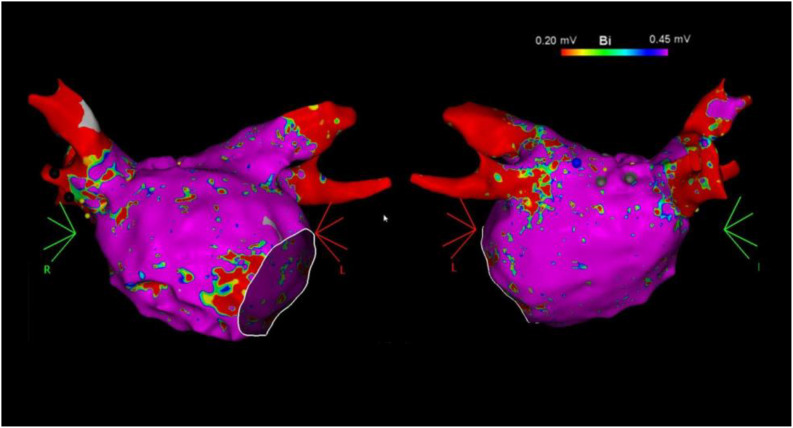
Example of left atrial bipolar voltage maps (0.20–0.45 mV) in the anterior **(left)** and posterior **(right)** views recorded with the multielectrode catheter prior to catheter ablation.

Our technique of AF mapping and ablation has been previously described (Squara et al., 2015; Liang et al., 2017). Briefly, 7 Fr- decapolar catheters were advanced into the coronary sinus and posterior right atrium along the crista terminalis. A 64-element phased-array intracardiac echocardiography (ICE) catheter (AcuNav, ACUSON, Mountain View, CA, United States) was used to assist for transseptal access, catheter manipulation, and real-team monitoring of complications. Intravenous heparin with a target activated clotting time of >350 s was administered before obtaining left atrial access. Two transseptal punctures were performed through which the ablation and a multipolar mapping catheter with 2-6-2 inter electrode spacing (PentaRay, Biosense Webster) were advanced into the left atrium. ME-FAMs were created for each patient during sinus rhythm, using CARTO 3 (Biosense Webster) prior to ablation (Figure 1).

Atrial electrograms were captured by setting the window of interest as the cycle length of sinus rhythm and using the proximal coronary sinus electrogram as a reference. Only patients with detailed LA mapping with >1000 points uniformly distributed were included in the study. Points were acquired in the auto-freeze mode if the stability criteria in space (6 mm) and local activation time (0.5 ms) were met. Mapping was performed with an equal distribution of points using a fill-threshold of 5–10 mm. Routinely, the regions adjacent to the PVs including the posterior wall, the roof between the superior veins, and the anterior aspect of the PV antrum were more densely sampled. Intracardiac echocardiography, orthogonal fluoroscopy, electrogram characteristics (far field appearing signals), and a dedicated impedance-based tissue proximity algorithm (TPI, Biosense Webster) were utilized to monitor for adequate contact. Signals were filtered at 30–400 Hz and displayed at 100 mm/s.

The PVI was performed by an experienced operator with coronary sinus and/or right ventricular pacing under general anesthesia, with high frequency low volume (JET) ventilation to optimize stability, and a target contact force of 10–25 gm. Ablation was performed utilizing an open irrigated contact force catheter (Thermocool SmartTouch SF, Biosense Webster) routinely with wide antral circumferential ablation with a power of 30–40 W for 30 s anteriorly and 30 W for 10 to 15 s posteriorly while carefully monitoring electrogram amplitude change and impedance. An esophageal temperature probe was used during ablation on the posterior wall with careful monitoring of the temperature and power/duration titration when any increase in temperature was observed. All ablation lesions were labeled as 3 mm VisiTags and the interlesion distance was <5 mm with no visible gap on electroanatomic map. First pass isolation was considered as entrance and exit block of a PV after completion of index wide antral circumferential ablation around the vein.

### LA Regional Electrogram Voltages

**FIGURE 2:**
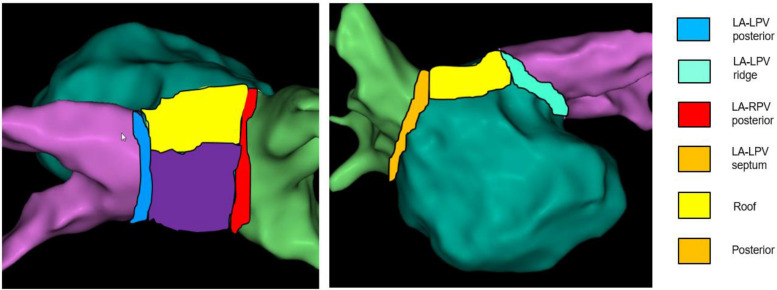
Regionalization of the left atrium. Schematic of six regions of pulmonary vein antrum sampled for voltage comparisons is provided.

Left atrial electrogram voltages were reviewed offline from ME-FAM maps obtained prior to the administration of any ablation lesions during the index procedure. To compare the regional voltage characteristics, the LA adjacent to the pulmonary veins was divided into six distinct regions (Figure 2). These regions included the posterior wall between the PV antrum, the adjacent LA roof, the posterior wall adjacent to the left pulmonary veins (Post LPV), the LPV ridge, the posterior wall adjacent to the right pulmonary vein (Post RPV), and the LA septum adjacent to the septal RPV. Each region extended approximately 5–10 mm in width. All points with ME-FAM in each of the LAs were collected and the mean voltage with standard deviation was calculated for each region. The site of difficult isolation was identified based on a review of the electroanatomic maps. All the bipolar electrogram signal amplitudes at sites (∼ 0.5–1.0 cm^2^) of difficult isolation during the initial mapping and prior to ablation were assessed and mean voltage was calculated for comparison with mean voltage for the remaining points in that LA region. Based on prior work (Sanders et al., 2003; Kapa et al., 2014), a voltage cutoff of <0.5 mV was considered abnormal and consistent with increase LA fibrosis.

### Statistical Analysis

Patient clinical and demographic characteristics were compared using χ^2^ or *t*-tests, as appropriate. Results are reported as percentages for categorical variables or means ± standard deviations for continuous variables. Mean LA segments voltages were calculated using all abstracted voltages in ME-FAM maps for both groups (Group 1: First pass isolation and Group 2: No first pass isolation). Paired *t*-tests were used to compare mean segment voltages between the two groups. All statistical analyses were performed using IBM SPSS Statistics 26.0 (IBM Corp, Armonk, NY, United States). All *P*-values were 2-sided with a significance threshold of 0.05.

## Results

### Patient Characteristics

Sixty consecutive patients who underwent catheter ablation for paroxysmal AF were screened. Of these, 20 patients (10 in each group; Group 1: First pass isolation and Group 2: No first pass isolation) had detailed ME-FAM obtained during sinus rhythm over all six regions and were included in the analysis. The baseline patient characteristics are summarized in Table 1 and were comparable in two groups. Only the mean age was greater in patients with first pass isolation (65.4 years in group 1 versus 61.2 years in group 2; *p* = 0.026).

**TABLE 1:** Patients’ baseline characteristics at the time of ablation.

**Characteristics**	**First pass (*n* = 10)**	**No first pass (*n* = 10)**	***p*-value**
Age (years)	65.4 ± 4.4	61.2 ± 11.3	0.026
Male (%)	7 (70%)	6 (60%)	0.639
BMI (kg/m^2^)	29.3 ± 3.2	30.2 ± 6.2	0.119
Paroxysmal AF	5 (50%)	5 (50%)	1
CHADSVASC	1.7 ± 1.3	2.4 ± 1.4	0.851
HTN	4 (40%)	6 (60%)	0.371
Diabetes	1 (10%)	2 (20%)	0.136
OSA	2 (20%)	1 (10%)	0.531
CAD	2 (20%)	3 (30%)	0.606
Stroke	0 (0%)	1 (10%)	0.305
CHF	2 (20%)	3 (30%)	0.606
Atrial flutter ablation	5 (50%)	6 (60%)	0.653
Mean LVEF	55.0 ± 9	55 ± 7	0.279
LA diameter (cm)	4.2 ± 0.7	4.7 ± 0.9	0.459

### Segmental Bipolar Voltage Characteristics and Site of Difficult Isolation

A total of 1986 electrograms were sampled in patients with first pass isolation compared to 2984 electrograms in the group without first pass isolation during sinus rhythm. The number of points sampled per LA region is summarized in Table 3. The mean segmental voltage characteristics are summarized in Table 2. Mean bipolar voltages obtained with ME-FAM were lower in all 6 segments in patients without first pass isolation compared to patients with first pass isolation; posterior wall (1.93 ± 1.46 versus 2.99 ± 2.19; *p* < 0.001), roof (1.83 ± 2.29 versus 2.47 ± 1.99; *p* < 0.001), LA-LPV posterior (1.85 ± 3.09 versus 2.99 ± 2.19, *p* < 0.001), LA-LPV ridge (1.42 ± 1.04 versus 1.91 ± 1.61; *p* < 0.001), LA-RPV posterior (1.51 ± 1.11 versus 2.30 ± 1.77, *p* < 0.001), and LA-RPV septum (1.55 ± 1.23 versus 2.31 ± 1.40, *p* < 0.001). The patients without first pass isolation also had a higher percentage of low voltage electrograms (<0.5 mV) in all 6 LA regions as depicted in Table 3.

**TABLE 2:** Comparison of mean segmental bipolar voltage in two groups.

**Segment**	**First pass isolation**	**No first pass**	***p-*value**
Posterior wall	2.99 ± 2.19	1.93 ± 1.46	<0.001
Roof	2.47 ± 1.99	1.83 ± 2.29	<0.001
RPV-septum	2.31 ± 1.40	1.55 ± 1.23	<0.001
LPV-ridge	1.91 ± 1.61	1.42 ± 1.04	<0.001
LPV posterior	2.5 ± 4.7	1.85 ± 3.09	<0.001
RPV posterior	2.30 ± 1.77	1.51 ± 1.11	<0.001

**TABLE 3:** Number of electrograms in each region and comparison of percentage of low voltage electrograms (<0.5 mV) based on ability to achieve first pass isolation.

	**First pass isolation**		**Without first pass isolation**	
	**Number of electrograms (Mean ± SD)**	**Low voltage electrograms**	**Number of electrograms (Mean ± SD)**	**Low voltage electrograms**
Posterior wall	68 ± 51	49/685 (7.1%)	93 ± 70	103/933 (11.0%)
Roof	27 ± 21	15/263 (5.7%)	46 ± 40	66/451 (14.6%)
LA-RPV septum	28 ± 17	17/281 (6.0%)	35 ± 23	64/348 (18.4%)
LA-RPV posterior	20 ± 7	22/311 (7.0%)	47 ± 36	54/395 (13.6%)
LA-LPV ridge	26 ± 15	22/193 (11.4%)	39 ± 14	95/473 (20.0%)
LA-LPV posterior	31 ± 22	25/253 (9.9%)	40 ± 26	46/384 (12.0%)

**FIGURE 3:**
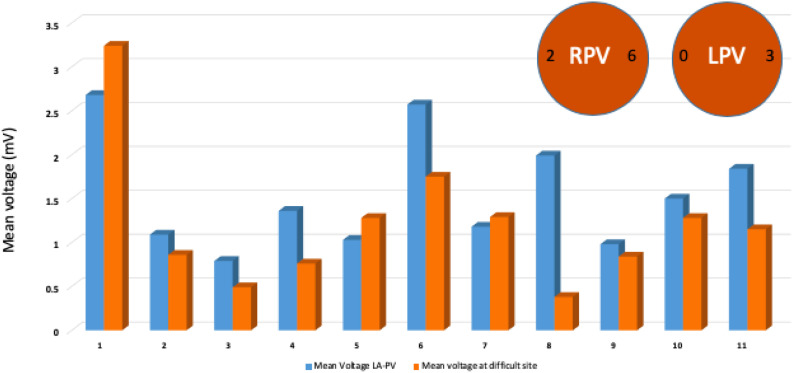
Mean voltage at the site of failure of first pass isolation compared to mean voltage at the remaining sites from same LA-PV junction region. Descriptive analysis and comparison performed and illustrated by bar graphs of mean voltage showing lower baseline voltage at 8 of 11 sites of persistent PV connections. Typical location of persistent PV connection at posterior or anterior carina after first pass isolation highlighted by circles.

Out of 10 patients who had no first pass isolation, the site of difficult isolation was located on the RPVs in eight patients (Posterior carina in 6 and septal carina in 2) and the LPVs were reconnected in three patients (posterior carina/roof). The mean voltage at the site of the difficult isolation was numerically lower compared to mean voltage for rest of same region at 8 of the 11 sites (Figure 3).

## Discussion

In patients undergoing initial catheter ablation for pAF, we documented that patients without first pass isolation have lower mean left atrial electrogram voltage as well as more scar burden as indexed by the percent signals <0.5 mV compared to patients who had first pass isolation. Mean voltage at the site of a difficult isolation was also typically lower compared to the mean voltage for the rest of the electrograms in that region. These observations have clinical implications and suggest that indices of global and segmental increases in fibrosis may identify patients with more difficulty achieving first pass isolation with radiofrequency ablation. This came as somewhat of a surprise because we had originally believed that more difficult to isolate segments might actually have larger amplitude signals because of the associated increase in LA thickness or muscle bundle size.

Pulmonary vein isolation is the most effective ablation strategy for AF management. With advances in technology and experience with catheter ablation, durability of PVI has improved over the past decade. However, PV reconnection continues to be a common occurrence accounting for the majority of recurrent AF ablation procedures. First pass isolation, defined as an entrance and exit block of a PV after the completion of wide antral circumferential ablation around the vein appears to be a reliable index for achieving long-term chronic PV isolation. Various techniques such as ablation index, contact force, overlapping lesions to minimize inter lesion distance, closing visual gaps in lesion sets, etc., have been proposed to increase the rate of first pass isolation (Taghji et al., 2018). When first pass isolation is not achieved, further mapping and ablation of the persistent PV connection site can be challenging due to local edema from prior ablation lesions. Various factors such as catheter stability, respiratory variations, power delivered, and tissue characteristics affect optimal lesion delivery. In this context, low atrial bipolar voltage as a marker of left atrial fibrosis is proposed as a potential contributor that may affect the biophysics of energy delivery during RFA.

Specific voltage thresholds to define atrial scarring have not been histologically validated (Sim et al., 2019). In a study of 20 patients with paroxysmal AF, Kapa et al. (2014) evaluated thresholds for atrial scarring and reported different cut offs for different segments of the left atrium. A threshold of <0.2 mV for the posterior wall and pulmonary vein/left atrial junctions, and <0.45 mV for all other atrial segments correlated best with scarring defined by late gadolinium enhancement on cardiac magnetic resonance imaging. In a study of atrial voltage in non-AF patients presenting for left sided accessory pathway ablation, Lin et al. reported a threshold of 0.4 mV to represent low voltage (Lin et al., 2014). However, these studies were performed with point by point mapping using a 3.5 mm bipolar electrode at the tip of an ablation catheter. In the current era of multipolar mapping, voltage thresholds to define abnormal substrate vary considerably. Liang et al. (2017) compared point by point mapping to ME-FAM in assessing left atrial voltage in patients presenting for repeat AF ablation, using a voltage cut off of <0.2 mV to define a dense scar. In that study, mean voltage in multiple segments of the left atrium (septum, posterior wall, right PV-LA junction) were significantly higher with ME-FAM compared to point by point mapping (Liang et al., 2017). Higher mapping resolution with multipolar catheters such as PentaRay (Biosense Webster) has been shown to identify higher voltage segments within an area of scarring (Anter et al., 2015).

In this relatively small number of patients, we also noted that difficulty in achieving first pass isolation is more common in the RPVs compared to LPVs, with the RPV carina being the most common site of difficulty. The RPVs have also been shown to have higher rates of spontaneous and provoked reconnection after achieving first pass isolation in prior studies (Andrade et al., 2020). Importantly, patients with acute PV reconnections also have lower arrhythmia-free survival long-term. The RPV carina presents a particularly challenging region during PVI. The presence of an epicardial connection between the RPV carina and the RA has been postulated as a potential mechanism of difficulty to achieve successful ablation (Yoshida et al., 2019). The septopulmonary bundle which is a broad band of epicardial fibers tends to connect at the RPV carina. Sites of difficult ablation of the RPV in our study showed that mean voltage at the site of difficult isolation along the RPVs was significantly lower compared to mean bipolar voltage of the remaining sites in that region segment, pointing toward scar/fibrosis in these regions that impede energy delivery at least on the posterior aspect of the carina. Early identification of these sites on sinus rhythm voltage could help direct energy delivery targeting higher ablation indices in these regions and more redundancy of lesions. This hypothesis will require additional investigation.

### Limitations

The cohort size for our study was relatively small and included only patients presenting for first time catheter ablation for pAF and may not apply to patients with persistent forms of AF. All maps were created in sinus rhythm; the results might not be reproducible if voltage maps are created in AF or atrial flutter. There was heterogeneity and large standard deviation in LA-LPV posterior despite adequate sampling. Some of this variability may be related to the gaps in myocardium noted posteriorly. The sampling was greater in patients without first pass isolation, however, low voltage regions were diffuse and involved all regions. We do not routinely assess for late gadolinium enhancement (LGE) with pre-ablation cardiac MRI, therefore we were unable to make comparisons between ME-FAM derived low voltage regions versus LA scar as seen on CMR in this patient series. We have previously reported a correlation between LGE on CMR and voltage <0.5 mV with point by point mapping (Kapa et al., 2014). Further studies are required to compare an LA scar identified by ME-FAM and LGE abnormalities on CMR. Finally, we only included cases performed with PentaRay catheter in this series, our findings may not apply to LA voltage abnormalities identified using other multipolar mapping catheters or the larger tipped ablation catheter.

## Conclusion

This study established that lower global LA voltage, and more low voltage signals (<0.5 mV), which suggest an increase in LA fibrosis, may identify patients with anticipated difficulty in achieving first pass isolation using radiofrequency ablation. Antral PV segments with difficult first pass isolation appear to be associated with lower rather than higher signal amplitude. The identification of relative low voltage areas could potentially guide optimal ablation energy delivery to achieve durable first pass isolation. Additional study is required to determine if there is a global voltage threshold effect which identifies risk and whether energy titration in higher risk patients can be used to improve the likelihood of first pass isolation and its relationship to long term success.

## Data Availability Statement

The raw data supporting the conclusions of this article will be made available by the authors, without undue reservation.

## Ethics Statement

The studies involving human participants were reviewed and approved by the University of Pennsylvania Health System’s Institutional Review Board. The patients/participants provided their written informed consent to participate in this study.

## Author Contributions

LG, NP, and FM contributed to the design and implementation of the project, analysis of the results, and writing of the final manuscript. MH, JA, CT, and PS contributed in interpreting the results and worked on the manuscript. All authors discussed the results and commented on the manuscript.

## Conflict of Interest

The authors declare that the research was conducted in the absence of any commercial or financial relationships that could be construed as a potential conflict of interest.

## Abbreviations

PVI: pulmonary vein isolation
AF: atrial fibrillation
ME-FAM: multielectrode fast automated mapping
LA: left atrium
LPV: left pulmonary vein
RPV: right pulmonary vein
CMR: cardiac magnetic resonance
LGE: late gadolinium enhancement.

## References

[B1] Andrade J. G. Deyell M. W. Nattel S. Khairy P. Dubuc M. Champagne J. (2020). Prevalence and clinical impact of spontaneous and adenosine-induced pulmonary vein reconduction in the Contact-Force vs. Cryoballoon atrial fibrillation ablation (CIRCA-DOSE) study. *Heart Rhythm.* 17 897–904. 10.1016/j.hrthm.2020.01.017 31978593

[B2] Anter E. Tschabrunn C. M. Josephson M. E. (2015). High-resolution mapping of scar-related atrial arrhythmias using smaller electrodes with closer interelectrode spacing. *Circ. Arrhythm. Electrophysiol.* 8 537–545. 10.1161/circep.114.002737 25792508

[B3] Callans D. J. Gerstenfeld E. P. Dixit S. Zado E. Vanderhoff M. Ren J. F. (2004). Efficacy of repeat pulmonary vein isolation procedures in patients with recurrent atrial fibrillation. *J. Cardiovasc. Electrophysiol.* 15 1050–1055. 10.1046/j.1540-8167.2004.04052.x 15363079

[B4] January C. T. Wann L. S. Alpert J. S. Calkins H. Cigarroa J. E. Cleveland J. C. Jr., (2014). AHA/ACC/HRS guideline for the management of patients with atrial fibrillation: a report of the American College of Cardiology/American Heart Association Task Force on Practice Guidelines and the Heart Rhythm Society. *J. Am. Coll. Cardiol.* 64 e1–e76. 24685669 10.1016/j.jacc.2014.03.022

[B5] Kapa S. Desjardins B. Callans D. J. Marchlinski F. E. Dixit S. (2014). Contact electroanatomic mapping derived voltage criteria for characterizing left atrial scar in patients undergoing ablation for atrial fibrillation. *J. Cardiovasc. Electrophysiol.* 25 1044–1052. 10.1111/jce.12452 24832482

[B6] Lemola K. Hall B. Cheung P. Good E. Han J. Tamirisa K. (2004). Mechanisms of recurrent atrial fibrillation after pulmonary vein isolation by segmental ostial ablation. *Heart Rhythm.* 1 197–202. 10.1016/j.hrthm.2004.03.071 15851153

[B7] Liang J. J. Elafros M. A. Muser D. Pathak R. K. Santangeli P. Supple G. E. (2017). Comparison of left atrial bipolar voltage and scar using multielectrode fast automated mapping versus point-by-point contact electroanatomic mapping in patients with atrial fibrillation undergoing repeat ablation. *J. Cardiovasc. Electrophysiol.* 28 280–288. 10.1111/jce.13151 27997060

[B8] Lin Y. Yang B. Garcia F. C. Ju W. Zhang F. Chen H. (2014). Comparison of left atrial electrophysiologic abnormalities during sinus rhythm in patients with different type of atrial fibrillation. *J. Interv. Card. Electrophysiol.* 39 57–67. 10.1007/s10840-013-9838-y 24113851

[B9] Natale A. Raviele A. Al-Ahmad A. Alfieri O. Aliot E. Almendral J. (2010). Venice Chart International Consensus document on ventricular tachycardia/ventricular fibrillation ablation. *J. Cardiovasc. Electrophysiol.* 21 339–379. 20082650 10.1111/j.1540-8167.2009.01686.x

[B10] Nilsson B. Chen X. Pehrson S. Kober L. Hilden J. Svendsen J. H. (2006). Recurrence of pulmonary vein conduction and atrial fibrillation after pulmonary vein isolation for atrial fibrillation: a randomized trial of the ostial versus the extraostial ablation strategy. *Am. Heart J.* 152 537.e1–537.e8. 16923426 10.1016/j.ahj.2006.05.029

[B11] Saini A. Huizar J. F. Tan A. Koneru J. N. Ellenbogen K. A. Kaszala K. (2017). Scar Homogenization in Atrial Fibrillation Ablation: Evolution and Practice. *J. Atr. Fibrillation.* 10:1645. 10.4022/jafib.1645 PMC5725749 29250241

[B12] Sanders P. Morton J. B. Davidson N. C. Spence S. J. Vohra J. K. Sparks P. B. (2003). Electrical remodeling of the atria in congestive heart failure: electrophysiological and electroanatomic mapping in humans. *Circulation* 108 1461–1468. 10.1161/01.cir.0000090688.49283.67 12952837

[B13] Sandorfi G. Rodriguez-Manero M. Saenen J. Baluja A. Bories W. Huybrechts W. (2018). Less pulmonary vein reconnection at redo procedures following radiofrequency point-by-point antral pulmonary vein isolation with the use of contemporary catheter ablation technologies. *JACC Clin. Electrophysiol.* 4 1556–1565. 10.1016/j.jacep.2018.09.020 30573119

[B14] Sim I. Bishop M. O’Neill M. Williams S. E. (2019). Left atrial voltage mapping: defining and targeting the atrial fibrillation substrate. *J. Interv. Card. Electrophysiol.* 56 213–227. 10.1007/s10840-019-00537-8 31076965 PMC6900285

[B15] Squara F. Frankel D. S. Schaller R. Kapa S. Chik W. W. Callans D. J. (2014). Voltage mapping for delineating inexcitable dense scar in patients undergoing atrial fibrillation ablation: a new end point for enhancing pulmonary vein isolation. *Heart Rhythm.* 11 1904–1911. 10.1016/j.hrthm.2014.07.027 25064249

[B16] Squara F. Liuba I. Chik W. Santangeli P. Maeda S. Zado E. S. (2015). Electrical connection between ipsilateral pulmonary veins: prevalence and implications for ablation and adenosine testing. *Heart Rhythm.* 12 275–282. 10.1016/j.hrthm.2014.11.003 25460169

[B17] Taghji P. El Haddad M. Phlips T. Wolf M. Knecht S. Vandekerckhove Y. (2018). Evaluation of a strategy aiming to enclose the pulmonary veins with contiguous and optimized radiofrequency lesions in paroxysmal atrial fibrillation: a pilot study. *JACC Clin. Electrophysiol.* 4 99–108. 10.1016/j.jacep.2017.06.023 29600792

[B18] Verma A. Wazni O. M. Marrouche N. F. Martin D. O. Kilicaslan F. Minor S. (2005). Pre-existent left atrial scarring in patients undergoing pulmonary vein antrum isolation: an independent predictor of procedural failure. *J. Am. Coll. Cardiol.* 45 285–292. 10.1016/j.jacc.2004.10.035 15653029

[B19] Yamaguchi J. Takahashi Y. Yamamoto T. Amemiya M. Sekigawa M. Shirai Y. (2020). Clinical outcome of pulmonary vein isolation alone ablation strategy using visitag surpoint in non-paroxysmal atrial fibrillation. *J. Cardiovasc. Electrophysiol.* 31 2592–2599. 10.1111/jce.14673 32666561

[B20] Yoshida K. Baba M. Shinoda Y. Harunari T. Tsumagari Y. Koda N. (2019). Epicardial connection between the right-sided pulmonary venous carina and the right atrium in patients with atrial fibrillation: a possible mechanism for preclusion of pulmonary vein isolation without carina ablation. *Heart Rhythm.* 16 671–678. 10.1016/j.hrthm.2018.11.017 30465905

